# Phytohormone Profile of *Medicago* in Response to Mycorrhizal Fungi, Aphids, and Gibberellic Acid

**DOI:** 10.3390/plants11060720

**Published:** 2022-03-08

**Authors:** Drew Olson, Hannah M. Berry, Jamie D. Riggs, Cristiana T. Argueso, Susana Karen Gomez

**Affiliations:** 1 School of Biological Sciences, University of Northern Colorado, Greeley, CO 80639, USA; drew.olson@usda.gov; 2 Toxicology & Mycotoxin Research Unit, United States Department of Agriculture, Agricultural Research Service, United States National Poultry Research Center, Athens, GA 30605, USA; 3 Department of Agricultural Biology, Colorado State University, Fort Collins, CO 80523, USA; hmberry@rams.colostate.edu (H.M.B.); Cris.Argueso@colostate.edu (C.T.A.); 4 School of Professional Studies, Northwestern University, Evanston, IL 60208, USA; jamie.riggs@northwestern.edu

**Keywords:** mycorrhizal symbiosis, pea aphids, phytohormones, plant susceptibility

## Abstract

Although gibberellic acid (GA) is widely used in agriculture, it is unclear whether exogenous GA makes aphid-infested, mycorrhizal plants more susceptible to herbivory. This study investigates the role of GA in modulating defenses in barrel medic plants (*Medicago truncatula*) that are infested with pea aphids (*Acyrthosiphon pisum*) and colonized by the beneficial symbiont *Rhizophagus intraradices*. Mock- and *R. intraradices*-inoculated potted plants were grown in a topsoil: sand mix for 42 days and were treated with GA or solvent. Subsequently, plants were exposed to herbivory or no aphid herbivory for 36 h and 7 days. Afterwards, plant growth parameters, aphid fitness, and foliar phytohormone concentrations were measured. The results revealed that GA regulates plant defenses during arbuscular mycorrhizal (AM) fungus–plant–aphid interactions as aphids that fed for 7 days on mycorrhizal, GA-untreated plants weighed more than those that fed on mycorrhizal, GA-treated plants. No major differences were detected in phytohormone levels at 36 h. Overall, mycorrhizal plants showed more shoot biomass compared to non-mycorrhizal controls. The arbuscule density and fungal biomass of *R. intraradices* were not altered by exogenous GA and aphid herbivory based on molecular markers. This study indicates that exogenous GA may help reduce aphid fitness when feeding on mycorrhizal plants.

## 1. Introduction

Soil microorganisms such as arbuscular mycorrhizal (AM) fungi form relationships with approximately 80% of plant roots, providing essential nutrients in exchange for carbohydrates and lipids from host plants [1,2,3,4,5,6]. AM fungi indirectly interact with above- and below-ground organisms by ‘priming’ plants for future engagement with pathogenic microbes and insect herbivores. This priming response appears to be regulated by jasmonic acid (JA) and can spread through extraradical mycorrhizal networks [7,8,9,10,11,12,13,14]. Even though AM fungi can prime plants for future insect attacks, certain insects such as aphids can benefit from feeding on mycorrhizal plants [15,16,17,18,19,20,21]. Most aphids feed on the phloem and are considered to be agriculturally important pests because they serve as vectors of approximately 275 plant viruses, leading to significant crop losses, especially in temperate regions [22,23]. Aphids use specialized mouthparts, stylets, to promote plant susceptibility by injecting saliva containing proteins and other compounds [24,25,26,27,28,29,30]. Aphid feeding on mycorrhizal plants results in above-ground changes in volatile compounds and foliar chemistry as well as below-ground changes in AM fungal root colonization and composition, plant carbon allocation to the AM fungus, and plant–plant communication via mycorrhizal networks [31,32,33,34,35,36,37,38].

For the most part, plant defenses are regulated by the antagonistic interactions between JA and salicylic acid (SA) [39,40]. In general, JA signaling promotes the synthesis of metabolites and proteins that induce resistance against insects and necrotrophic pathogens [40,41], while SA signaling induces defenses against biotrophic pathogens [42]. Nevertheless, aphids manipulate plant defenses by altering the balance between JA and SA [24]. The molecular mechanisms aphids use to manipulate plant defenses in pairwise interactions are complex and multifaceted [25]; however, these mechanisms in tripartite interactions are poorly understood. Nonetheless, other phytohormones are involved in modulating JA and SA signaling to prioritize plant defense and growth responses. For instance, gibberellic acid (GA) dictates many plant growth and developmental processes and also modulates JA signaling and defense responses [43]. GA signaling is repressed by nuclear GRAS domain repressor proteins known as DELLAs [43]. GA is perceived by the receptor GIBBERELLIN INSENSITIVE DWARF 1 (GID1), forming a GA–GID1 complex that further interacts with and signals for DELLA degradation via the 26S proteasome pathway [43,44]. In addition, DELLAs can act as transactivating factors for JA signaling by relieving the master regulator MYC2 from JASMONATE ZIM-domain (JAZ) repressor proteins; therefore, GA signaling serves a negative role for JA signaling through DELLA degradation [45]. It has been further demonstrated that DELLAs confer resistance to necrotrophic pathogens, while decreasing resistance to biotrophic pathogens [45,46]. The silencing of phytoene desaturase (PDS), an enzyme involved in GA biosynthesis, leads to the increased systemic accumulation of an aphid-born virus, the Potato Leaf Roll Virus (PLRV) [47]. Interestingly, the fecundity of the green peach aphid (*Myzus persicae*) improved when they fed on PDS-silenced plants relative to PLRV-infected plants [47]. GA signaling and the modulation of JA signaling via DELLAs has been characterized in a few plant–pathogen interactions [45,46]; however, the role of GA signaling in insect–plant interactions seems to be context dependent and is mostly unknown in insect–plant–beneficial microbe interactions.

For example, exogenous GA application negatively affects rice (*Oryza sativa*) resistance to root-knot nematodes (*Meloidgyne graminocola*) through DELLA degradation and decreased JA signaling [48]. Additionally, DELLA proteins have been shown to be important for the beet armyworm (*Spodoptera exigua*) labial saliva-related weaking of defense responses in *Arabidopsis* [49]. Conversely, there is evidence of a positive role of GA signaling in mediating resistance against the brown planthopper (*Nilparvata lugens*) [50]. However, Chen and coworkers indicate that GA-mediated resistance may have been attributed to other mechanisms such as increased lignification [50]. On the other hand, DELLA proteins are key components of a transcriptional network to positively regulate AM symbioses [51,52]. Gibberellin biosynthesis and perception appear to negatively regulate AM symbioses through DELLA degradation [51,53,54,55,56]. The present study builds on our previous data which showed that pea aphid (*Acyrthosiphon pisum*) feeding in shoots leads to the upregulation of bioactive GA biosynthesis (GA2-oxidase) gene expression in mycorrhizal *M. truncatula* roots [57]. This study investigates the role of exogenous GA application to roots in modulating *M. truncatula* defenses during interactions with pea aphids and the AM fungus *Rhizophagus intraradices*. We predicted that (a) exogenous GA and/or high levels of root colonization by AM fungi would stimulate plant growth and would positively affect aphid fitness (7 days), (b) exogenous GA and/or aphid herbivory would reduce arbuscule formation, and (c) exogenous GA, root colonization by AM fungi, and/or aphid herbivory would lead to altered levels of foliar phytohormones. The experiment included GA- and solvent-treated, mock- and *R. intraradices*-inoculated potted plants that were subsequently exposed to 36 h and 7 days of herbivory or no aphid herbivory. Plant growth parameters, aphid fitness, and foliar phytohormone concentrations were measured when the feeding period concluded.

## 2. Results

### 2.1. Shoot and Root Fresh Weight after 36 h of Pea Aphid Feeding

**Figure 1:**
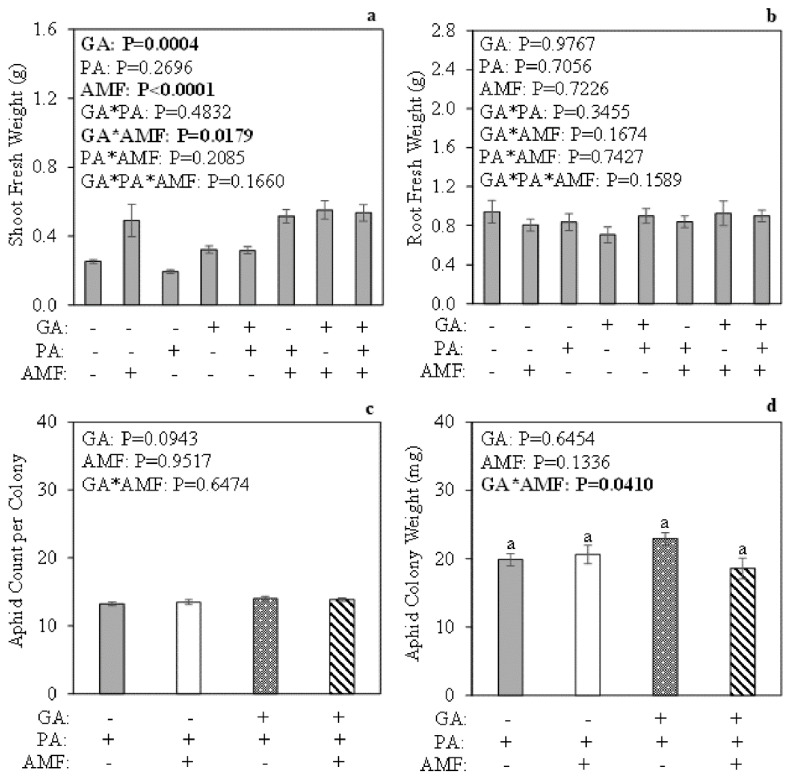
Impact of exogenous gibberellic acid (GA) application, 36 h of pea aphid (*Acyrthosiphon pisum*) (PA) feeding, and AM fungus (*Rhizophagus intraradices*) (AMF) root colonization on *Medicago truncatula* (**a**) shoot and (**b**) root growth. Impact of exogenous GA and AM fungus root colonization on (**c**) aphid count per colony and (**d**) aphid colony weight. Potted plants were grown in topsoil: sand mix for 44 days. Plants were harvested at 51 days post seed sterilization. Three-factor analysis using gamma distribution (**a**), three-factor ANOVA (**b**), two-factor analysis using Poisson distribution with scaled Pearson χ2 (**c**), and two-factor ANOVA (**d**) were used. Values represent the mean ± SEM (*n* = 7 or 8) per treatment. Different letters represent significant differences among groups using Tukey–Kramer post hoc tests (*p* < 0.05).

To determine if exogenous GA application and/or root colonization by AM fungi stimulated plant growth, fresh weight measurements were taken prior to freezing plant tissues. At 36 h post aphid feeding, the interaction among exogenous GA, aphid herbivory, and AM fungus root colonization (GA*PA*AMF) did not have a significant effect on shoot fresh weight (Figure 1a; *p* = 0.1660). However, the interaction between exogenous GA and AM fungal colonization (GA*AMF) regardless of aphid herbivory status (+/−PA) had a significant effect on shoot fresh weight (Figure 1a; *p* = 0.0179). The post hoc analysis revealed that mycorrhizal plants had the highest fresh shoot weight compared to the other treatments (Figure S1). On the other hand, root fresh weight was not significantly affected by exogenous GA, pea aphid feeding, and AM fungus root colonization (Figure 1b; *p* = 0.1589).

### 2.2. Pea Aphid Count and Colony Weight at 36 h Post Feeding

To assess if exogenous GA application and/or root colonization by AM fungi impacted pea aphid performance after 36 h of feeding, pea aphids were collected, counted, and weighed. The interaction between exogenous GA and AM fungus root colonization (GA*AMF) did not have a significant impact on aphid count per colony (Figure 1c; *p* = 0.6474), but the interaction between exogenous GA and AM fungus root colonization (GA*AMF) had a significant impact on aphid colony weight (Figure 1d; *p* = 0.0410); however, a post hoc analysis showed no detectable differences among the treatment groups (Figure 1d).

### 2.3. Phytohormone Profile in Medicago Leaves at 36 h Post Aphid Feeding

To determine if exogenous GA application, root colonization by AM fungi, and/or aphid herbivory would lead to altered levels of phytohormones, their profile was assessed on *Medicago* leaves via ultra-performance liquid chromatography-mass spectrometry (UPLC-MS). This time point was chosen based on previous research on *Medicago*–pea aphid interactions [58]. The phytohormones that were quantified include: abscisic acid (ABA), dihydrophaseic acid (DPA), indole-3-acetic acid (IAA), indole-3-acetyl alanine (IA alanine), indole-3-acrylic acid (IAcrA), indole-3-acetonitrile (IA nitrile), indole-3-butyric acid (IBA), indole-3-carboxylic acid (ICA), jasmonic acid (JA), phaseic acid (PA), methyl salicylate (meSA), salicylic acid (SA), *trans*-zeatin (tZ), and *trans*-zeatin riboside (tZR).

**Figure 2:**
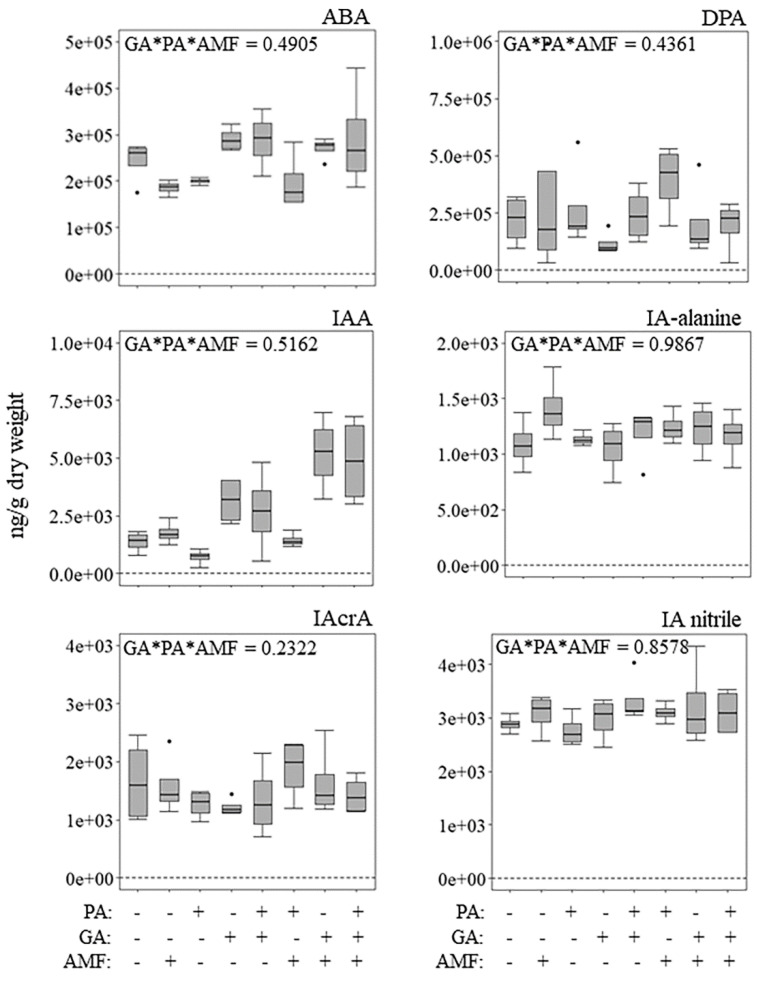

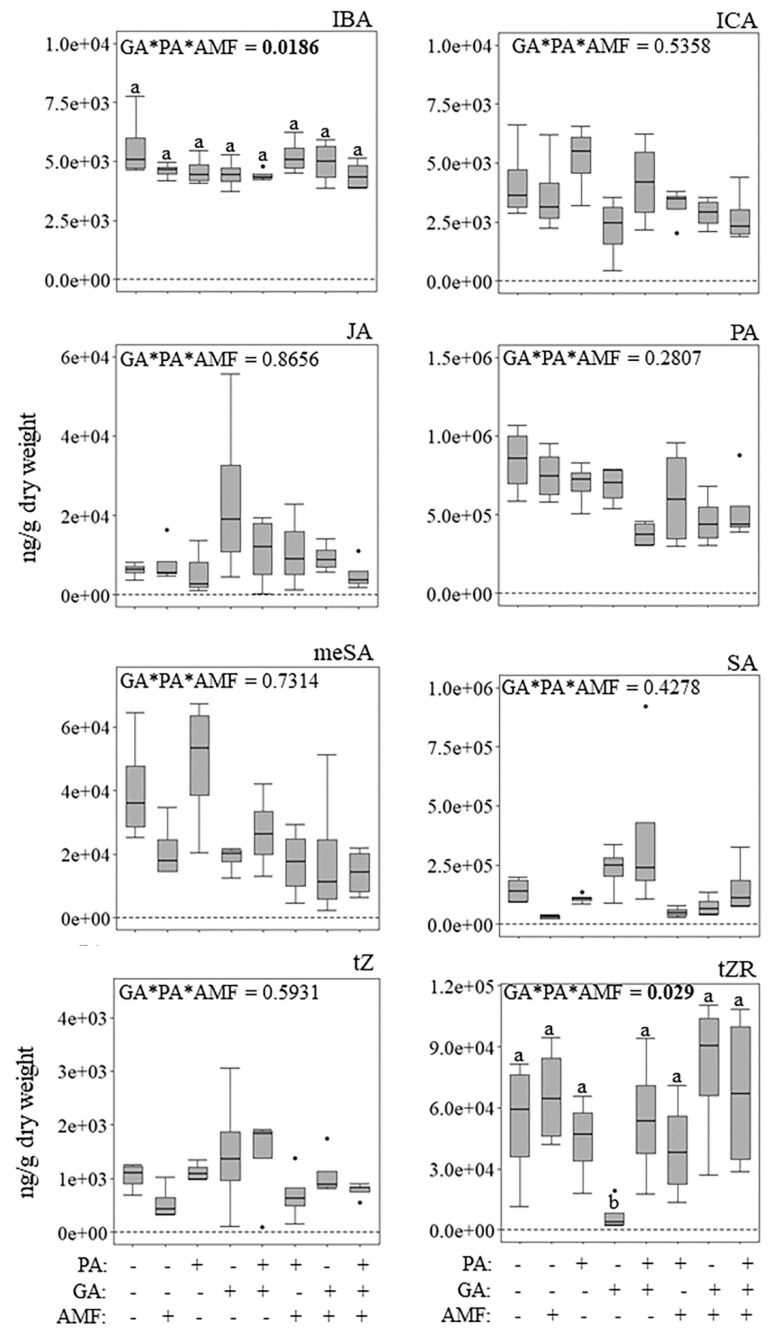
Impact of exogenous gibberellic acid (GA) application, 36 h of pea aphid (*Acyrthosiphon pisum*) (PA) feeding, and AM fungus (*Rhizophagus intraradices*) (AMF) root colonization on phytohormone concentration in lamina and petiole tissue. Potted plants were grown in topsoil: sand mix for 44 days. Plants were harvested at 51 days post seed sterilization. Three-factor analysis using gamma distribution was used. Values represent the mean ± SEM (*n* = 4). Different letters represent significant differences among groups using Tukey–Kramer post hoc tests (*p* < 0.05). ABA = abscisic acid; DPA = dihydrophaseic acid; IAA = indole-3-acetic acid; IA alanine = indole-3-acetyl alanine; IAcrA = indole-3-acrylic acid; IA nitrile = indole-3-acetonitrile; IBA = indole-3-butyric acid; ICA = indole-3-carboxylic acid; JA = jasmonic acid; PA = phaseic acid; mesa = methyl salicylate; SA = salicylic acid; tZ = *trans*-zeatin; tZR = *trans*-zeatin riboside.

Overall, the interaction among exogenous GA, aphid herbivory, and AM fungus root colonization (GA*PA*AMF) did not have a significant effect on the concentration of most phytohormones tested at 36 h post aphid feeding (Figure 2; Table S1). The only significant interactions (GA*PA*AMF) were observed for IBA and tZR (Figure 2; Table S1). However, the post hoc analysis for IBA did not detect differences among treatment groups, probably due to small sample size (*n* = 4). In contrast, the post hoc analysis for tZR showed that plants treated with GA only (+GA − PA − AMF) had the lowest concentration of tZR relative to the other treatments (Figure 2).

The only two-factor interaction (GA*AMF) that was statistically significant was for JA (Table S1; *p* = 0.0261). However, the post hoc analysis did not detect statistically significant differences among treatments (Figure S2). The only comparison that was close to being statistically significant (*p* = 0.0588) was for GA untreated, non-mycorrhizal plants with or without aphids (−GA −/+PA − AMF) versus GA-treated, non-mycorrhizal plants with or without aphids (+GA −/+ PA − AMF) (Figure S2). The main effect AM fungus root colonization (AMF) was significant for IAA, PA, meSA, SA and tZ, while the main effect aphid herbivory (PA) was only significant for SA (Table S1; *p* = 0.0378). The main effect exogenous GA was significant for ABA, IAA, ICA, meSA, and SA (Table S1).

### 2.4. Mycorrhizal Root Markers at 36 h Post Aphid Feeding

To determine if exogenous GA application and/or aphid herbivory would reduce arbuscule formation after 36 h of aphid feeding, the gene expression of a plant phosphate transporter (*MtPT4*) gene that is expressed exclusively in root cells with arbuscules and the *R. intraradices* elongation factor (*RiEF*) gene that provides information about fungal biomass were measured. The interaction between exogenous GA and aphid herbivory (GA*PA) and the main effects were not statistically significant for *RiEF* relative gene expression (Figure S3). The interaction between exogenous GA and aphid herbivory (GA*PA) had a significant effect on *MtPT4* relative gene expression in roots (Figure S3; *p* = 0.0335); however, a post hoc analysis did not detect statistically significant differences among the treatment groups (Figure S3). The only comparison that was close to being statistically significant (*p* = 0.0560) was for non-infested, GA-untreated, mycorrhizal plants (−GA − PA + AMF) versus aphid-infested GA-untreated, mycorrhizal plants (−GA + PA + AMF).

### 2.5. Shoot and Root Fresh Weight after 7 Days of Aphid Feeding

**Figure 3:**
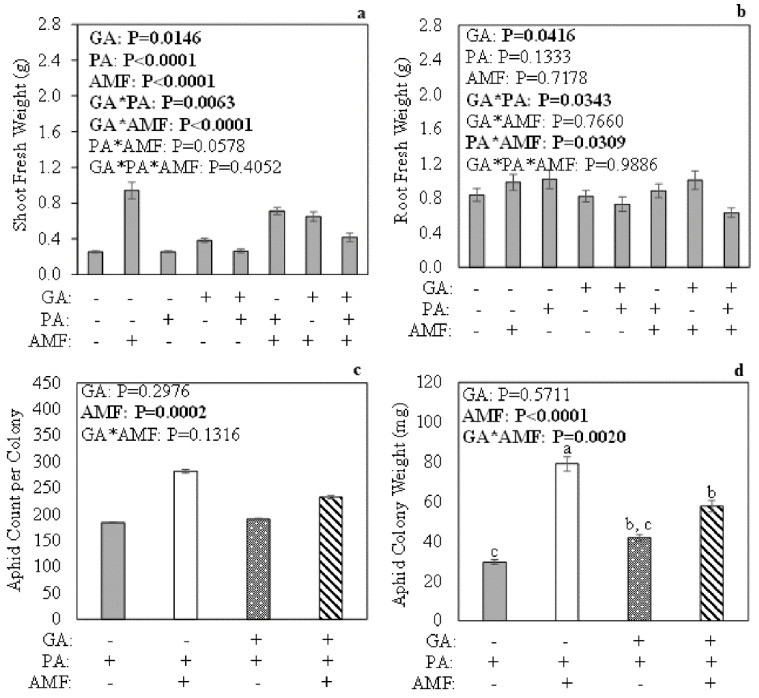
Impact of exogenous gibberellic acid (GA) application, 7 days of pea aphid (*Acyrthosiphon pisum*) (PA) feeding, and AM fungus (*Rhizophagus intraradices*) (AMF) root colonization on *Medicago truncatula* (**a**) shoot and (**b**) root growth. Impact of exogenous GA and AM fungus root colonization on (**c**) aphid count per colony and (**d**) aphid colony weight. Potted plants were grown in topsoil: sand mix for 49 days. Plants were harvested at 56 days post seed sterilization. Three-factor analysis using gamma distribution (**a**), three-factor ANOVA (**b**), two-factor analysis using Poisson distribution with scaled Pearson χ2 (**c**), and two-factor ANOVA (**d**) were used. Values represent the mean ± SEM (*n* = 6 to 8) per treatment. Different letters represent significant differences among treatment groups using Tukey–Kramer post hoc tests (*p* < 0.05).

To determine if aphid feeding for an extended period, exogenous GA application, and root colonization by AM fungi alter plant growth, fresh weight measurements were taken prior to freezing plant tissues. Overall, the interaction among exogenous GA, aphid herbivory, and AM fungus root colonization (GA*PA*AMF) did not have a significant effect on shoot fresh weight 7 days post aphid feeding (Figure 3a; *p* = 0.4052). The interaction between exogenous GA and aphid herbivory (GA*PA), regardless of AM status (−/+AMF), had a significant effect on shoot fresh weight (Figure 3a; *p* = 0.0063). Aphid-infested, GA-untreated plants regardless of AM status (+GA + PA −/+ AMF) accumulated less shoot fresh weight compared to the other treatments (Figure S4a). Additionally, the interaction between exogenous GA and AM fungus root colonization (GA*AMF), regardless of aphid herbivory (+/−PA), did have a significant effect on shoot fresh weight (Figure 3a; *p* < 0.0001). A post hoc analysis revealed that all the treatments were statistically significant from each other (Figure S4b). Overall, mycorrhizal plants accumulated more shoot fresh weight (Figure S4b).

The interaction among exogenous GA, aphid herbivory, and AM fungus root colonization (GA*PA*AMF) did not have a significant effect on root fresh weight 7 days post aphid feeding (Figure 3b; *p* = 0.9886). The interactions of exogenous GA and aphid herbivory (GA*PA), regardless of AM fungus root colonization (+/−AMF), had a significant effect on root fresh weight. A post hoc analysis showed that fresh roots of GA-treated, aphid-infested plants (+GA + PA −/+ AMF) weighed less compared to the GA untreated, aphid-infested plants (−GA +PA −/+ AMF) (Figure S4c). Additionally, the interaction between aphid herbivory and AM fungus root colonization (PA*AMF) regardless of exogenous GA (+/−GA) had significant effects on root fresh weight 7 days post aphid feeding (Figure 3b); however, the post hoc analysis did not detect differences among treatments (Figure S4d).

### 2.6. Pea Aphid Count Per Colony and Colony Weight at 7 Days Post Feeding

To determine if exogenous GA application and/or high colonization levels by AM fungi would positively affect aphid fitness, aphids were collected, counted, and weighed. The interaction between exogenous GA and AM fungus root colonization (GA*AMF) had a significant effect on aphid colony weight 7 days post aphid feeding (Figure 3d; *p* = 0.0020). The main effect root colonization by AM fungi (AMF) was also statistically significant for both aphid count per colony and aphid colony weight (Figure 3c,d). 

Aphid colony weight was higher for aphids that fed continuously for 7 days on plants that were colonized by AM fungi without receiving GA (−GA + PA + AMF) compared to the other treatment groups, whereas this fitness parameter had lower values for aphids that fed on non-mycorrhizal plants without receiving GA (−GA + PA − AMF) (Figure 3d). However, aphid colony weights for aphids that fed continuously for 7 days on non-mycorrhizal, GA-untreated plants (−GA + PA − AMF) did not differ from aphids that fed on non-mycorrhizal plants that received GA (+GA + PA − AMF). In addition, aphid colony weight for aphids that fed continuously for 7 days on mycorrhizal plants that received GA (+GA + PA + AMF) did differ from aphids that fed on non-mycorrhizal only (−GA + PA − AMF) (Figure 3d).

## 3. Discussion

In the present study, we investigated the role of exogenous GA application to roots in modulating *M. truncatula* susceptibility during interactions with pea aphids and the AM fungus *R. intraradices*. To our knowledge, no study involving this type of three-way interaction has been reported, so this research paves the way for future research involving complex interactions that may occur in natural settings. Two aphid feeding time-points were used, 36 h and 7 days. The 36 h time-point was mainly used to examine the early changes in phytohormone levels that could lead to an impact on aphid fitness (7 days).

As expected, aphid abundance and colony weight were not drastically impacted by the interaction between exogenous GA and AM fungus root colonization after 36 h of feeding (Figure 1c,d). Overall, mycorrhizal plants accumulated more shoot biomass, followed by non-mycorrhizal, GA-treated plants, whereas non-mycorrhizal, GA-untreated plants accumulated the least (Figure S1). The results after 7 days of aphid feeding showed that the interaction between exogenous GA and AM fungus colonization, regardless of aphid herbivory status, had an impact on shoot fresh weight (Figure S4b). Overall, mycorrhizal plants accumulated more shoot biomass compared to non-mycorrhizal plants (Figure S4b), which agrees with previous *M. truncatula* data [59,60]. Interestingly, aphids that fed for 7 days on mycorrhizal, GA-untreated plants weighed more than aphids that fed on mycorrhizal, GA-treated plants, whereas aphids that fed on non-mycorrhizal, GA-untreated plants weighed the same as aphids that fed on non-mycorrhizal, GA-treated plants (Figure 3d), indicating that GA may regulate the balance between defense versus plant growth [61,62]. There are reports of negative impacts on different aphid species when plants are treated with foliar GA alone or in combination with other plant hormones [63,64]. Studies that tested GA on insects found no significant effects on fall armyworm larvae when they fed on plants treated with foliar GA [65,66,67]. However, there is evidence showing that GA can be cytotoxic against the melon fruit fly (*Bactrocera cucurbitae*) (Coquillett) [68], different types of Lepidopteran larvae, and migratory locust (*Locusta migratoria migratoria*) [69,70,71,72]. Gibberellic acid also negatively affects fall armyworm (*S. frugiperda*) food consumption and female oviposition [73,74]. The data also agree with previous studies showing that pea aphids benefit from feeding on plants that are highly colonized by AM fungi compared to non-mycorrhizal control plants [20,32,57]. Even though the data did not support the prediction that aphid fitness would be positively impacted by exogenous GA application to mycorrhizal plants, the results indicate that GA plays a role in AM fungus–plant–aphid interactions and may be worth pursuing in future studies.

We detected a few statistically significant three-factor interaction (GA*PA*AMF) differences in phytohormone concentration after 36 h of aphid feeding (Figure 2). Two possible reasons could be the feeding time-point and perhaps the pea aphid clone used in this study. Our chosen time-point was based on previous pea aphid-*M. truncatula* data [58]. Stewart and coworkers measured JA, SA and ABA concentrations at 0, 12, 24 and 48 h post pea aphid feeding, and found increased levels of these phytohormones at 24 and 48 h post-feeding [58]. They also found different plant responses depending on the pea aphid clone that was used (PS01 and N116). Future studies involving three factors should consider examining additional aphid feeding time-points to have a more complete picture of the plant responses. The only phytohormone that showed a statistically significant three-factor interaction was *trans*-zeatin riboside (tZR) (Figure 2). Non-mycorrhizal, non-aphid-infected plants treated with GA (−PA + GA − AMF) showed the least concentration of tZR compared to all other treatments.

Interestingly, relative gene expression for an arbuscule-specific marker (*MtPT4*) was not significantly impacted by exogenous GA and aphid herbivory (Figure S3), indicating that once arbuscules developed in a root system, they are unaffected by these treatments. Previous research has found that exogenous GA treatment starting at 6 days post inoculation (dpi) prevents arbuscule development in plant roots [51], and that pea aphid feeding can negatively impact the AM fungus colonization of roots [32]. In addition, we used the *RiEF* gene to quantify and compare fungal biomass across treatments. Although fungal biomass was not significantly impacted by exogenous GA in this study, this may be due to hyphal growth throughout the root length. Previous research has shown that the AM fungal phenotype in plants that received exogenous GA treatment at 6 dpi is the same as in *Mtdella1/Mtdella2* mutant plants. In both cases, arbuscule development was severely decreased, while there were no differences between wild type and *Mtdella1/Mtdella2* in root length colonization [51]. This may be due to increased hyphal branching, which was found to be increased by exogenous GA treatment, while arbuscules were nearly undetectable [51]. There were several differences between the present study and the study by Floss and coworkers [51]. In the present study, GA treatments were started at 7 dpi and *R. intraradices* was used. We inoculated plants that were 22 days old, whereas previous research inoculated 2-day-old plants [51]. It is worth pointing out that exogenous GA affects Arum- and Paris-type AMs differently [75]. Future studies could examine the impact of exogenous GA comparing different Arum-type species. Additionally, AM fungi (*R. irregularis*) can produce phytohormones, including GA [76]. To determine the impact of exogenous GA on arbuscule development during tripartite interactions, future studies should consider coupling molecular markers with measurements of arbuscule density via microscopy [51]. 

In conclusion, the present study revealed that GA may play a role in the regulation of plant defenses during AM fungi–plant–aphid interactions. The data did not provide support to our hypothesis that aphid fitness improves when feeding on mycorrhizal plants treated with exogenous GA. Finally, the data showed that exogenous GA did not affect *R. intraradices* arbuscule density or fungal biomass based on plant and fungal molecular markers. This study serves as a focal point for future research seeking to elucidate the complex role of GA in regulating plant defenses in AM fungus–plant–aphid interactions.

## 4. Materials and Methods

### 4.1. Medicago truncatula Sterilization, Germination, and Growth Conditions

Seed sterilization and germination procedures for *M. truncatula* Jemalong A17 followed a protocol outlined in Maurya et al. [57]. Seeds were initially scarified in concentrated H_2_SO_4_ for 10 min, rinsed in sterile water three times, sterilized for 10 min using 10% (*v*/*v*) household bleach in 0.1% (*v*/*v*) Tween 20 solution, and rinsed in sterile water five times [57]. Immediately following sterilization, seeds were spread on wet autoclaved filter paper in Petri dishes, which were sealed with parafilm and covered in aluminum foil. Dishes were incubated in a 4 °C refrigerator for three days, kept at room temperature (24 °C) for one day (dark), and then the aluminum foil was removed and dishes were placed under indirect light (mean: 184 µmol m^−2^ s^−1^) for three days. Seedlings were transplanted into sterilized mason sand: topsoil mix (9:1) (Pioneer Sand Company, Windsor, CO, USA) in azalea pots (12 cm W × 8.5 cm H), and were grown in a PGC-flex growth chamber (Conviron Inc., Pembina, ND, USA) under a 16 h photoperiod with the following conditions: 24 °C, 40% relative humidity, and light intensity of 285–290 µmol m^−2^ s^−1^, provided by fluorescent and halogen light bulbs. The sand was thoroughly washed with tap water and the topsoil was sieved (sieve no. 8) prior to autoclaving both substrates three times (each cycle: 60 min, 121 °C, at 15 psi). The washed/autoclaved sand: topsoil mix was sent to Weld Laboratories, Inc. (Greeley, CO, USA) for soil analysis. The mixture had the following characteristics: pH 7.3, 0.17% organic matter, 6.8 ppm nitrate, 3 ppm phosphorus, 23 ppm potassium, 464 ppm calcium, 255 ppm sulfate, 0.8 ppm boron, 10.8 ppm iron, 3.2 ppm manganese, 0.6 ppm copper, and 0.42% estimated carbon. The sand: topsoil mix was saturated with ½ strength modified Hoagland’s solution (100 µM P, 15 mM N, pH 6.1) prior to transplant [77]. Azalea pots were covered by clear plastic humidity domes (54.6 cm H × 28 cm W × 17.8 cm D) for one week after transplant. Once domes were removed, plants were watered daily with 50 mL of Milli-Q^®^ water or received fertilizer treatment twice a week with 50 mL ½ strength modified Hoagland’s solution (100 µM P, 15 mM N, pH 6.1).

### 4.2. Rhizophagus intraradices Inoculation

Fifteen days post transplant into azalea pots, each *M. truncatula* seedling with two or three trifoliolate leaves was subsequently transplanted into an individual pot (6.35 cm W × 9 cm H) with either a 1:10 dilution of *R. intraradices* inoculum or a 1:10 dilution of mock inoculum (root exudates without AM fungi). Mock and *R. intraradices* (UT118, IA506, and Co204) inocula were purchased from the International Culture Collection of (Vesicular) Arbuscular Mycorrhizal Fungi (INVAM) (Morgantown, West Virginia, USA). The inoculation mixtures consisted of a 2.5 cm bottom layer of autoclaved sand:topsoil (9:1) substrate, followed by a 1.5 cm layer of a 1:10 *R. intraradices* inoculum or mock inoculum, and a 1.5 cm top layer of reused soil substrate from the seedlings’ azalea pots. Prior to transplanting, substrate or inoculum layers were moistened with ½ strength modified Hoagland’s solution (100 µM P, 15 mM N, pH 6.1). Immediately following inoculation, plants were covered by clear plastic humidity domes (54.6 cm H × 28 cm W × 17.8 cm D) for one week and were grown in a plant growth chamber for the remainder of the experiment. 

In addition, extra plants were inoculated to assess root colonization levels by *R. intraradices* prior to adding aphids to experimental plants. To assess the level of *R. intraradices* colonization, roots were cleared with 10% (*w*/*v*) KOH (85 °C for 4 h), rinsed with deionized water, and stained with 5% (*v*/*v*) Sheaffer black ink that was prepared in 5% (*v*/*v*) acetic acid [78]. After staining, *R. intraradices* colonization was quantified using the modified gridline-intersect method with the aid of an Olympus SZX10 stereo microscope (Leica Microsystems, Wetzlar, Germany) [79]. Plants receiving mock inoculum did not show staining for fungal structures. Aphids were added to experimental plants when *R. intraradices* colonization reached an average of 64% of root length colonization in the extra inoculated plants [57].

### 4.3. Exogenous GA_3_ Treatment

A 50 mg mL^−1^ GA_3_ stock solution (PhytoTechnology Laboratories, Lenexa, KS, USA) was made in 100% ethanol. A 10^−6^ M working solution was made daily from the stock solution using Milli-Q^®^ water [51]. One week after inoculation with AM fungi and mock inocula, plants received 17.5 mL of 1 µM GA_3_ or control treatments daily (except when fertilized). Plants were fertilized with 17.5 mL of ½ strength Hoagland’s solution (100 µM P, 15 mM N, pH 6.1) twice a week for the remainder of the experiment.

### 4.4. Pea Aphid Infestation

Parthenogenetic, wingless female pea aphids were reared on fava bean (*V. fava)* plants in insect tents in the laboratory under a 16 h photoperiod. One thousand six-day-old aphids [58] were synchronized by adding wingless adult aphids to a separate insect tent with non-infested fava beans. The adults were removed the following day, leaving only nymphs. Nymphs were reared on fava beans for an additional five days. After *R. intraradices* colonization reached the desired levels, 15 six-day-old aphids were added to each plant receiving aphids. Harvests took place between 10:00 and 16:00 h [80,81]. One replicate of every treatment was harvested at the same time to account for changes in plant responses that are regulated by circadian rhythms. The present study consisted of eight treatments with eight biological replicates each that are shown in Table 1.

Immediately after aphid infestation took place, all plants, including non-infested plants, were covered with organza drawstring gift bags (15.24 × 22.86 cm, SumDirect, Dongguan, Guangdong) and were rubberbanded. Aphids fed continuously on plants for 36 h [58] and 7 days [57]. At the end of each feeding period, aphids were gently collected from each plant using a vacuum device and were immobilized at −20 °C prior to counting and weighing them. Aphid weights were measured within one week of collection. Non-infested plants were also exposed to the vacuuming effect. The vacuum device consisted of a 4.8 mm diameter Tygon tubing, connected to a 50 mL conical tube (Fisher Scientific, Hampton, NH, USA), and a cut 200 µL pipette tip. Plants were harvested at 51 and 56 days post seed sterilization. Fresh weights for roots and shoots were measured using an analytical scale prior to freezing the tissue in liquid nitrogen and storage at −80 °C.

### 4.5. Effect of GA_3_ Treatment on Phytohormones during Aphid–Plant–AM Fungus Interactions

#### Phytohormone Quantification

Phytohormone extraction and concentration was determined using UPLC-MS and was performed only on leaves following the procedure outlined by Sheflin et al. [81] with some modifications. Plant shoot samples were stored at −80 °C post- harvest, lyophilized and subsequently extracted using a monophasic methyl-tert-butyl ether (MTBE) extraction protocol [81]. First, 19.4 mg of lyophilized leaf (blade and petiole) tissue was measured using an analytical balance and subsequently treated with 990 µL of HPLC grade MTBE and 10 µL of internal standard mix and vortexed at 4 °C for 2 h. Samples were centrifuged at 3500× *g* at 4 °C for 15 min, and 750 µL of supernatant was transferred to new microcentrifuge tubes to incubate overnight at −80 °C. Next, samples were centrifuged at 18,000× *g* at 4 °C for 20 min. Samples were dried under nitrogen conditions at room temperature and resuspended in 100 µL of HPLC-grade methanol and stored at −80 °C. Samples were sent to the Proteomics and Metabolomics Facility at Colorado State University, Fort Collins, CO where UPLC-MS was conducted [81]. Phytohormones in this method span five hormone classes and include: abscisic acid (ABA), dihydrophaseic acid (DPA), indole-3-acetic acid (IAA), indole-3-acetyl alanine (IA alanine), indole-3-acrylic acid (IAcrA), indole-3-acetonitrile (IA nitrile), indole-3-butyric acid (IBA), indole-3-carboxylic acid (ICA), jasmonic acid (JA), phaseic acid (PA), methyl salicylate (meSA), salicylic acid (SA), trans-zeatin (tZ), and trans-zeatin riboside (tZR).

### 4.6. Root RNA Isolation and cDNA Synthesis

RNA isolation and cDNA synthesis followed the protocol outlined by Rizzo et al. [82]. First, three biological replicates were selected randomly within each of the treatments for root gene expression analyses. Roots were ground separately using a mortar and pestle in liquid nitrogen. RNA was extracted using the RNeasy Plant Mini Kit following the manufacturer’s instructions (Qiagen Inc., Germantown, MD, USA). RNA samples were treated with 87 µL of nuclease-free water, 10 µL of 10X reaction buffer, and 3 µL of Turbo^TM^ DNase (2 units µL^−1^) for a total volume of 100 µL and incubated at 37 °C for 40 min. DNAse-treated RNA samples were purified using the RNeasy MinElute Cleanup kit (Qiagen Inc.). An additional DNAse treatment was performed using the DNA-free^TM^ DNA removal kit (Thermo Fisher Scientific, Waltham, MA, USA) following the manufacturer’s protocol for RNA samples exhibiting trace amounts of genomic DNA contamination. 

For cDNA synthesis, 1 µg of total RNA was mixed with 1 µL dNTPs (10 mM each) and 1 µL anchored oligo dT_22_ (500 ng µL^−1^) and incubated at 65 °C for 5 min using a T100™ Thermal Cycler (Bio-Rad Laboratories Inc., Hercules, CA, USA). Each sample was then brought to a total volume of 20 µL by adding 4 µL of SuperScript^®^ IV Buffer, 1.2 µL Nuclease-free water, 1 µL of DTT (100 µM), 0.5 µL of RNaseOUT^TM^ (Thermo Fisher Scientific), and 0.3 µL of SuperScript^®^ IV (Thermo Fisher Scientific). Samples were subsequently incubated at 50 °C for 10 min and 80 °C for 10 min using a T100™ Thermal Cycler (Bio-Rad Laboratories Inc.).

### 4.7. Assessment of Mycorrhizal Root Markers

Markers involved in arbuscule/fungus presence such as the *Medicago* phosphate transporter 4 gene (*MtPT4*) [83] and the AM fungal reference gene elongation factor (*RiEF*) [84] were used together with the *Medicago* reference gene glyceraldehyde-3-phosphate dehydrogenase (*GAPDH*) [85,86].

To assess gene expression, 1 µL of cDNA template (1:5), 5 µL of PowerSYBR^®^ Green Master Mix (Thermo Fisher Scientific), 2 µL of autoclaved Milli-Q^®^ water (Merck KGaA, Darmstadt, Germany), and 1 µL of 3 µM forward and reverse primers was used. Each of the 384-well plates were run on a C1000^®^ Touch ThermalCycler (Bio-Rad, Hercules, CA, USA) and each run included two technical replicates and 3–4 biological replicates per treatment. The thermal profile comprised an initial incubation at 95 °C for 10 min, followed by 40 cycles at 95 °C for 15 s, an annealing/extension at 53.3–63.3 °C for 1 min, and a melt curve analysis ranging from 65–95 °C that increases incrementally by 5 °C. Oligonucleotide sequences and annealing temperatures used for RT-qPCR are reported in Table S2. The 2^−ΔCq^ method was used to calculate relative gene expression [87].

### 4.8. Statistical Analyses

Statistical analyses were carried out using SAS 9.4 for Windows (SAS Institute Inc, Cary, NC, USA). Normal distribution of raw data was determined using Shapiro–Wilk and Anderson–Darling tests (*p* > 0.05). Three-factor analyses of variance (ANOVA) were used to determine the interaction effect of exogenous GA_3_ application, aphid herbivory, and AM fungus root colonization on plant growth parameters and phytohormone concentration. Data on plant growth exhibiting non-normal distribution were subsequently analyzed by three-factor analyses using log-linked gamma distributions. Three-factor analyses using log-linked gamma distributions were also used to determine the interaction effect of GA_3_ application, aphid herbivory, and AM fungus root colonization on phytohormone concentration. Two-factor ANOVAs were used to determine the effect of GA_3_ application and AM fungus root colonization on mean aphid colony weights. Aphid colony weight data that exhibited non-normal distribution were subsequently analyzed using two-factor analyses using log-linked gamma distributions. Mean aphid count per colony data were analyzed with two-factor analyses using counts models, Poisson or negative binomial distributions. In addition, two-factor ANOVAs were used to determine the effect of GA_3_ application and aphid feeding on mycorrhizal marker gene expression.

## Figures and Tables

**Table 1:** Experimental treatments used to investigate the role of exogenous application of gibberellic acid (GA) to *Medicago truncatula* that were inoculated with mock inoculum or *R. intraradices* inoculum and infested with pea aphids.

Treatment Order	Gibberellic Acid (GA) Applied	AM Fungi (AMF) Presence	Pea Aphid (PA) Presence
1	−	−	−
2	−	−	+
3	−	+	−
4	+	−	−
5	+	+	−
6	−	+	+
7	+	−	+
8	+	+	+

## Supplementary Materials

The following supporting information can be downloaded at https://www.mdpi.com/article/10.3390/plants11060720/s1. Figure S1: Impact of exogenous gibberellic acid (GA) application, 36 h of pea aphid (*Acyrthosiphon pisum*) (PA) feeding, and AM fungus (*Rhizophagus intraradices*) (AMF) root colonization on *Medicago truncatula* shoot growth. Potted plants were grown in topsoil: sand mix for 44 days. Plants were harvested at 51 days post seed sterilization. Two-factor ANOVA was used. Values represent the mean ± SEM. Different letters represent significant differences among groups using Tukey–Kramer post hoc tests (*p* < 0.05). Figure S2: Impact of exogenous gibberellic acid (GA) application, 36 h of pea aphid (*Acyrthosiphon pisum*) (PA) feeding, and AM fungus (*Rhizophagus intraradices*) (AMF) root colonization on phytohormone concentration in lamina and petiole tissue. Potted plants were grown in topsoil: sand mix for 44 days. Plants were harvested at 51 days post seed sterilization. Two-factor ANOVA was used. Values represent the mean ± SEM (*n* = 4). Different letters represent significant differences among groups using Tukey–Kramer post hoc tests (*p* < 0.05). JA = jasmonic acid. Figure S3: Impact of exogenous gibberellic acid (GA) application, 36 h of pea aphid (*Acyrthosiphon pisum*) (PA) feeding, and AM fungus (*Rhizophagus intraradices*) (AMF) root colonization on *RiEF* and *MtPT4* relative gene expression on mycorrhizal *Medicago truncatula* roots. Potted plants were grown in topsoil: sand mix for 44 days. Plants were harvested at 51 days post seed sterilization. The interaction between exogenous GA, and pea aphid feeding (GA*PA) had a statistically significant effect on *MtPT4* relative gene expression according to the two-factor ANOVA (*p* = 0.0335); however, no differences in *MtPT4* relative gene expression were observed among treatment groups according to Tukey–Kramer post hoc tests (*p* > 0.05). Values represent the mean ± SEM (*n* = 3 or 4). Figure S4. Impact of exogenous gibberellic acid (GA) application, 7 days of pea aphid (*Acyrthosiphon pisum*) (PA) feeding, and AM fungus (*Rhizophagus intraradices*) (AMF) root colonization on *Medicago truncatula* (a,b) shoot and (c,d) root growth. Potted plants were grown in topsoil: sand mix for 49 days. Plants were harvested at 56 days post seed sterilization. Two-factor ANOVA was used. Values represent the mean ± SEM. Different letters represent significant differences among treatment groups using Tukey–Kramer post hoc tests (*p* < 0.05). Table S1: Three-factor ANOVA for foliar phytohormones after 36 h of pea aphid herbivory. Table S2. Primers used for RT-qPCR.
