# Supplementary material for: Phytohormone Profile of Medicago in Response to Mycorrhizal Fungi, Aphids, and Gibberellic Acid
Source: Plants (Basel). 2022 Mar 8;11(6):720. doi: 10.3390/plants11060720 (PMC8951282; doi:10.3390/plants11060720)
Supplement: Supplementary file 1 [file plants-11-00720-s001.zip › Suppl Files/Table S1.pdf]

Table S1. Three-factor ANOVA for foliar phytohormones after 36 hours of pea aphid herbivory.

Hormone	GA	PA	AMF	GA*PA	GA*AMF	PA*AMF	GA*PA*AMF
ABA	**<0.0001**	0.7848	0.1802	0.4521	0.3879	0.1907	0.4905
DPA	0.0338	0.2581	0.1902	0.7385	0.5806	0.3320	0.4361
IAA	**<0.0001**	0.0591	**0.0007**	0.2512	0.8026	0.3214	0.5162
IA-alanine	0.3926	0.9320	0.0388	0.4756	0.3566	0.1449	0.9867
IAcrA	0.1320	0.5684	0.0898	0.7748	0.7414	0.5007	0.2322
IA-Nitrile	0.5003	0.2432	0.2641	0.4931	0.2204	0.1371	0.8578
IBA	0.1264	0.3244	0.7527	0.5545	0.3405	0.1312	**0.0186**
ICA	**0.0270**	0.2378	0.1475	0.3496	0.4216	0.0623	0.5358
JA	0.2309	0.3098	0.473	0.1459	**0.0261**	0.6160	0.8656
PA	0.5874	0.2132	**0.0002**	0.5412	0.3638	0.0661	0.2807
MeSA	**0.0372**	0.9495	**0.0052**	0.8992	0.1778	0.1769	0.7314
SA	**<0.0001**	**0.0378**	**<0.0001**	0.1390	0.6536	0.1381	0.4278
tZ	0.1057	0.9340	**0.0194**	0.4131	0.8139	0.8478	0.5931
tZR	0.1947	0.1538	0.0021	0.0039	0.0046	0.0043	**0.0293**
