# Supplementary material for: Phytohormone Profile of Medicago in Response to Mycorrhizal Fungi, Aphids, and Gibberellic Acid
Source: Plants (Basel). 2022 Mar 8;11(6):720. doi: 10.3390/plants11060720 (PMC8951282; doi:10.3390/plants11060720)
Supplement: Supplementary file 1 [file plants-11-00720-s001.zip › Suppl Files/Table S2.pdf]

Table S2. Primers used for RT-qPCR.

Gene	Primer sequence (5' – 3')	Average Annealing Temperature (°C)	Efficiency
GAPDH-F	AACATCATTCCCAGCAGCAA	56.60	94%
GAPDH-R	AACATCGACGGTAGGCACAC		
PT4-F	GACACGAGGCGCTTTCATAGCAGC	60.00	99%
PT4-R	GTCATCGCAGCTGGAACAGCACCG		
RiEF-F	GCTATTTTGATCATTGCCGCC	60.00	118%
RiEF-R	TCATTAAAACGTTCTTCCGACC		
