# Supplementary figures and images for: Phytohormone Profile of Medicago in Response to Mycorrhizal Fungi, Aphids, and Gibberellic Acid
Source: Plants (Basel). 2022 Mar 8;11(6):720. doi: 10.3390/plants11060720 (PMC8951282; doi:10.3390/plants11060720)

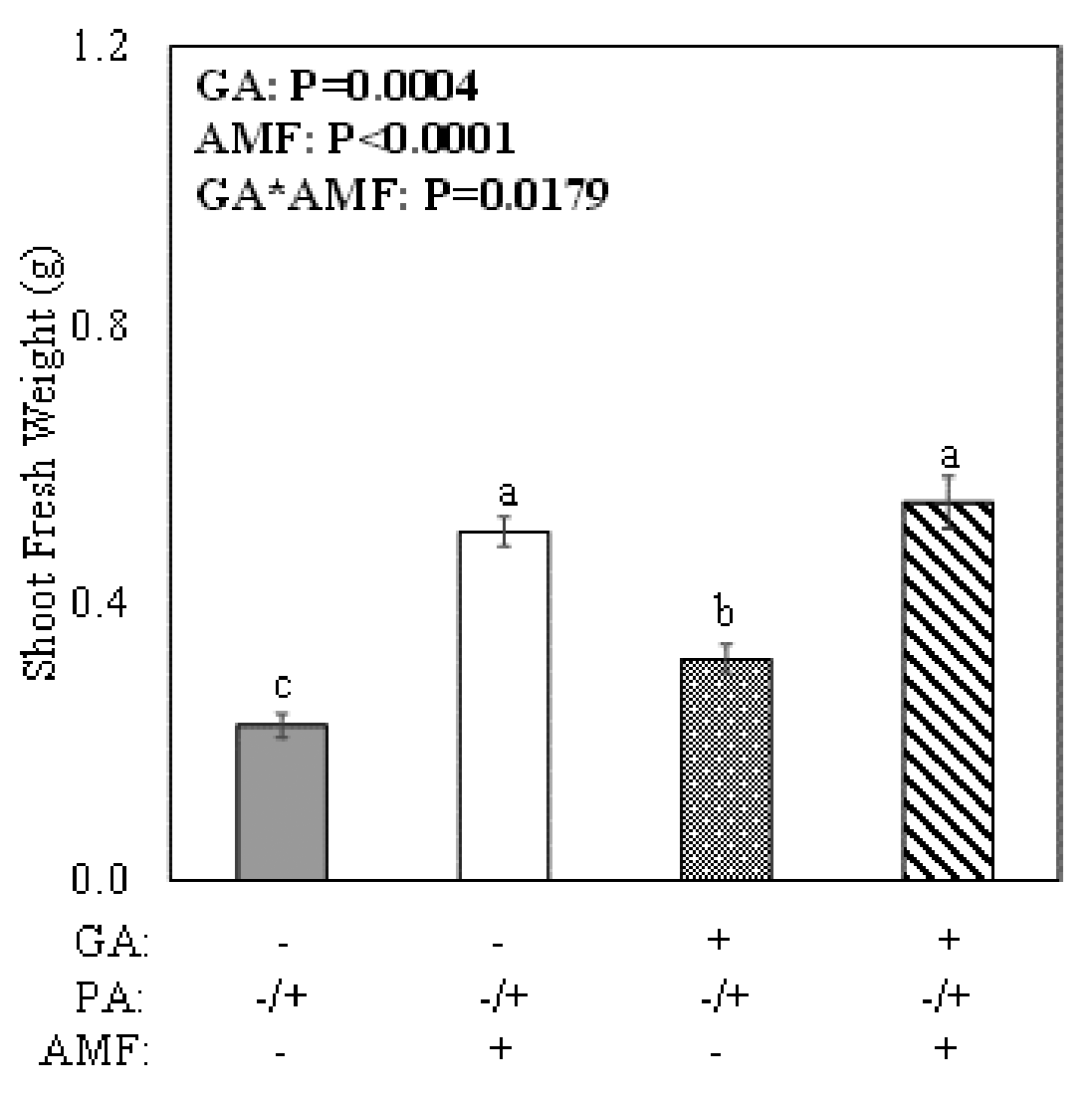

Supplement: Supplementary file 1 [file plants-11-00720-s001.zip › Suppl Files/Figure S1.tif]

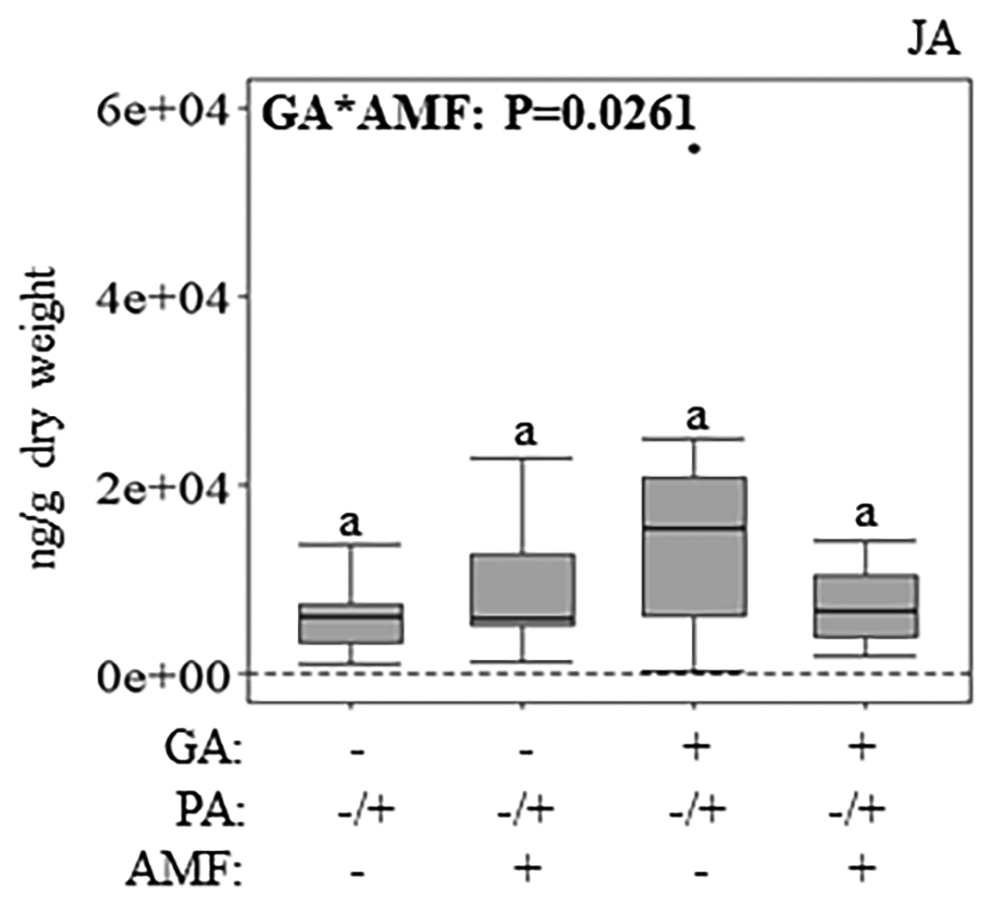

Supplement: Supplementary file 1 [file plants-11-00720-s001.zip › Suppl Files/Figure S2.tif]

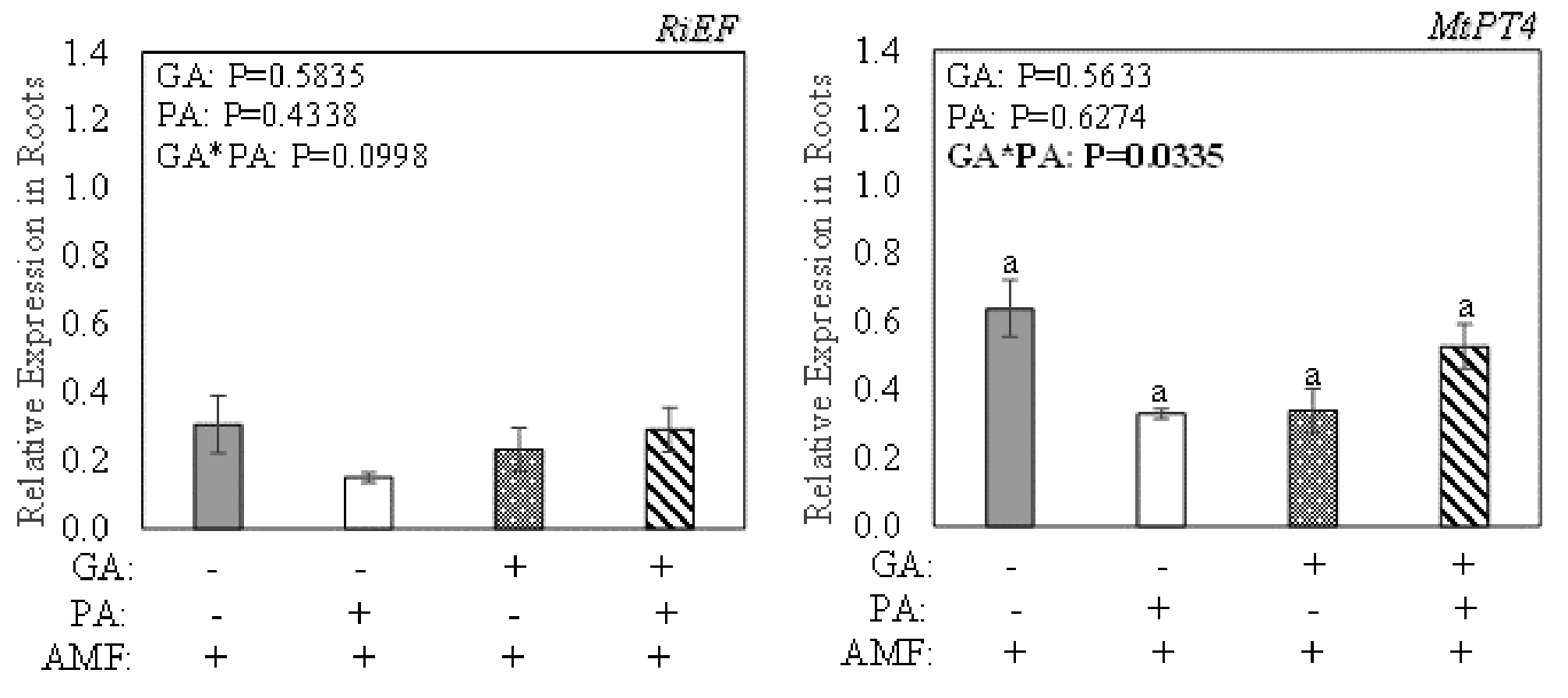

Supplement: Supplementary file 1 [file plants-11-00720-s001.zip › Suppl Files/Figure S3.tif]

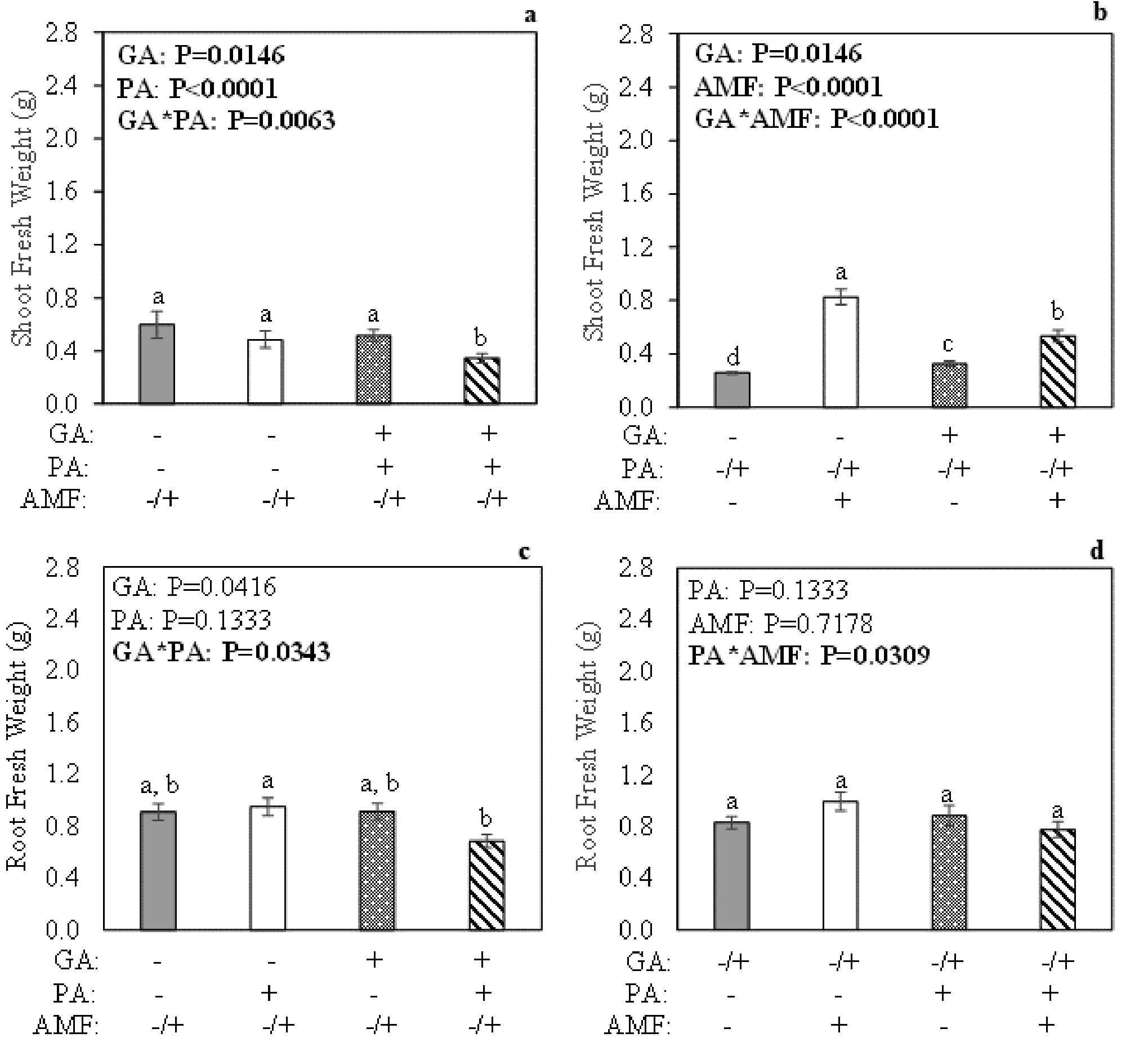

Supplement: Supplementary file 1 [file plants-11-00720-s001.zip › Suppl Files/Figure S4.tif]
